# Biological consequences of Z-nucleic acid sensing by ZBP1 and ADAR1

**DOI:** 10.1080/15476286.2026.2691476

**Published:** 2026-06-18

**Authors:** Fei Lu, Linghuan Tang, Chun Kim, Dengming Lai, Jinfa Tou, Huipeng Jiao

**Affiliations:** a Department of Neonatal Surgery, Children’s Hospital, Zhejiang University School of Medicine, National Clinical Research Center for Children and Adolescents’ Health and Diseases, Hangzhou, China; b Zhejiang Key Laboratory of Molecular Cancer Biology, Life Sciences Institute, Zhejiang University, Hangzhou, China; c School of Biopharmaceutical Convergence, Hanyang University [ERICA Campus], Ansan, Republic of Korea; d Zhejiang Key Laboratory of Neonatal Diseases, Hangzhou, China

**Keywords:** Z-nucleic acid, cell death, inflammation, Zα domain, ZBP1

## Abstract

Since their discovery, Z-nucleic acids (Z-NAs), which adopt a left-handed double helical conformation, have puzzled researchers regarding their physiological functions. These unusual nucleic acids are recognized by proteins containing Zα domains, particularly Z-DNA binding protein 1 (ZBP1) and adenosine deaminase acting on RNA 1 (ADAR1). Utilizing mouse genetics with knockout models and site-specific Zα domain mutations, scientists have revealed that Z-NAs serve as critical regulators of programmed cell death, inflammation, antiviral immunity, and anti-tumour responses. This review systematically examines mechanistic insights from *Zbp1*- and *Adar1*-mutant models, illuminating how Z-NAs play a dual role as essential triggers of host defence and as potential drivers of autoinflammatory diseases.

## Introduction

Nucleic acids, including deoxyribonucleic acid (DNA) and ribonucleic acid (RNA), are essential macromolecules that store and transmit genetic information, governing central biological processes. During infection, pathogen-derived nucleic acids function as pathogen-associated molecular patterns (PAMPs), engaging host pattern recognition receptors (PRRs) to orchestrate immune defences [1]. For instance, viral DNA is detected by dedicated cytosolic sensors such as cyclic GMP-AMP synthase (cGAS) and absent in melanoma 2 (AIM2), while viral RNA is recognized by sensors including retinoic acid-inducible gene I (RIG-I) and melanoma differentiation-associated protein 5 (MDA5). This recognition elicits potent antiviral and pro-inflammatory responses [1]. Beyond exogenous threats, abnormally accumulated self-nucleic acids can act as danger-associated molecular patterns (DAMPs) [2]. The recognition of these nucleic acids by specific sensor proteins underscores their pivotal roles in antiviral immunity, autoinflammatory disorders, and oncology.

Beyond their sequence-based functions, nucleic acids exhibit remarkable conformational polymorphism. Under physiological conditions, they primarily adopt canonical right-handed helices such as B-DNA, A-DNA, and A-RNA. However, under specific conditions, they can transition into left-handed conformations known as Z-DNA and Z-RNA, collectively termed Z-nucleic acids (Z-NAs) [3]. In recent years, the biological functions of Z-NAs have emerged as an area of intense scientific interest. Studies now demonstrate that Z-NAs play key roles in physiological and pathological processes, including cell death, antiviral immunity, and the pathogenesis of inflammatory diseases [3]. This review provides an overview of the history of Z-NA discovery and the cognate Zα sensing domains. We place a particular emphasis on how Z-NAs are regulated and sensed by Zα-containing proteins – most notably ZBP1 and ADAR1—detailing how these molecular events culminate in antiviral, inflammatory, autoinflammatory, and tumour-related outcomes.

## Discovery of Z-NAs

Since the seminal proposal of the DNA double-helix model by James Watson and Francis Crick in 1953 [4], our understanding of nucleic acid structure and function has evolved substantially. In this canonical model, DNA adopts a right-handed helical conformation known as B-DNA, the predominant form of double-stranded DNA (dsDNA) under physiological conditions [4]. B-DNA is characterized by its wide, deep major grooves and narrow, shallow minor grooves [5]. Notably, under dehydrating conditions, B-DNA can transition into A-DNA, another right-handed variant [6]. Unlike B-DNA, A-DNA features narrow, deep major grooves complemented by wide, shallow minor grooves [5].

In 1972, Fritz Pohl and Thomas Jovin reported a salt-induced conformational transition of poly(dG-dC), observing that high salinity (5 M NaCl) transforms the polymer from its standard B-form into an alternative helical structure [7]. This biochemical observation was structurally substantiated in 1979, when Alexander Rich and colleagues elucidated the crystal structure of the d(CGCGCG)_2_ fragment, uncovering a remarkable left-handed double helix characterized by Watson-Crick base pairing and antiparallel sugar-phosphate chains [8]. Unlike the uniform *anti*-conformation in B-DNA, this structure features alternating *anti*- and *syn*-glycosidic bonds, resulting in a characteristic zigzag backbone – hence the designation Z-DNA [8]. While B-DNA exhibits a smooth backbone with distinct grooves, Z-DNA possesses a nearly flat major groove and significantly deeper minor grooves [8]. Following the discovery of Z-DNA, a left-handed RNA double helix – poly(G-C) – was identified under conditions of high ionic strength, as evidenced by nuclear magnetic resonance (NMR), circular dichroism (CD), and absorbance spectroscopy [9]. In 2004, the Popenda group resolved the structure of Z-RNA r(CGCGCG)_2_, revealing that it closely resembles Z-DNA, with 12.4 base pairs per helical turn compared to 12 in Z-DNA [10]. While DNA readily adopts A-, B-, and Z-conformations, RNA predominantly exists in A- or Z-forms, rarely transitioning to the B-conformation.

The formation of Z-NAs exhibits distinct sequence preferences, typically requiring alternating purine-pyrimidine (APP) motifs. This requirement stems from the fact that purine glycosidic bonds more readily adopt the *syn*-conformation compared to pyrimidines, making APP sequences energetically favourable for the alternating *anti*–*syn* configuration [8]. Among these, poly(dG-dC) is the most canonical motif prone to Z-DNA transition. However, Z-DNA is thermodynamically unstable under physiological conditions, requiring a substantial energy barrier to transition from the B-form. Consequently, even sequences like poly(dG-dC) predominantly maintain a right-handed helix in a physiological environment. This intrinsic instability has made it difficult to understand the functional significance of Z-NAs as dynamic, conditional regulators.

While Z-DNA is thermodynamically unstable under physiological conditions, it can be stabilized in high-salt environments [11]. This is primarily because the phosphate groups in the Z-DNA backbone are in closer proximity than those in B-DNA, resulting in stronger electrostatic repulsion [12]. The addition of high concentrations of cations, particularly multivalent ones, mitigates this repulsion by neutralizing the negative charges. Although these high-salt requirements differ from the intracellular environment, endogenous polyamines – such as spermine, putrescine, and spermidine – can exert analogous stabilizing effects [13]. Furthermore, the incorporation of chemically modified nucleotides can energetically favour the *syn*-conformation. By imposing steric constraints or modulating base hydrophilicity, specific chemical modifications can stabilize poly(dG-dC) in the Z-conformation under physiological salinity. These include C8-bromination of guanine and C5-bromination of cytosine [14], N7 methylation of guanosine [15], 2’-O-methyl-8-methyl modification of guanosine (m^8^Gm) [16], C8 oxidation of guanosine [17], and substitution of cytosine with fluorinated arabinocytosine at the 2’ position and the fluorinated ribose at the 2’ position of guanine [18] (Table 1). Notably, the m^8^Gm modification also effectively maintains the Z-conformation in dsRNA [19] (Table 1). Beyond chemical modification, negative supercoiling provides a powerful biophysical mechanism to stabilize Z-DNA at physiological salt concentrations [20]. Leveraging this principle, ‘Z-B chimeras’ – stable circular constructs where left-handed dsDNA (Z) coexists with right-handed dsDNA (B) – were developed. The chimeras are stabilized via the topological restriction of short (60–120 nt) complementary DNA loops, which provides the requisite energy to maintain the non-canonical Z-conformation under physiological conditions [21]. Remarkably, this approach can stabilize even non-APP sequences in the Z-conformation [22]. Negative supercoiling, generated to relieve the topological strain of DNA unwinding during DNA replication and transcription, serves as a primary driver for Z-DNA formation in cells. This structural shift implies potential functional roles for Z-DNA *in vivo*. Indeed, Chromatin Immunoprecipitation (ChIP)-Seq profiling has confirmed the existence of Z-DNA within transcriptionally active regions, reinforcing its link to genomic regulation [23].

**Table 1.** Summary of nucleoside chemical modifications that stabilize the Z-NA conformation.

Modified nucleotides	Chemical structure	Example sequence
8-Bromo-2’-deoxyguanosine; 5-Bromocytidine	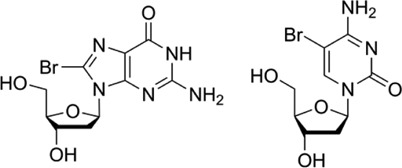	Brominated poly(dG-dC) [14]
7-Methyl-2’-deoxyguanosine (m^7^dG)	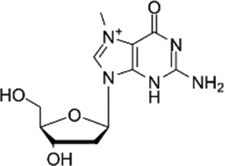	poly(m^7^dG-dC) [15]
2′-O-Methyl-8-methyl guanosine (m^8^Gm)	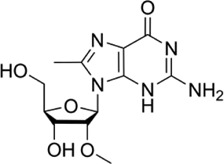	d(CGCm^8^GmCG)_2_ [16]; r(Cm^8^GmCm^8^GmCG)_2_ [19]
8-Hydroxy-2’-deoxyguanosine (8odG)	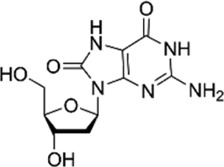	(8odGdCdGdC)_3_ [17]
2′-Deoxy-2′-fluoroguanosine (rfG); 2’-Fluoro-2’-deoxy-arabino-cytidine (afC)	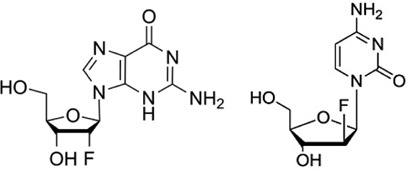	(afC-rfG)_10_ [18]

Brominated poly(dG-dC) has been employed as an antigen to generate Z-DNA-specific antibodies by immunizing animals [24]. Notably, the Z22 antibody generated by this strategy not only recognizes Z-DNA but also exhibits cross-reactivity with Z-RNA [25]. While X-ray diffraction, CD, and NMR have extensively elucidated these structures, early structural frameworks initially struggled to bridge the gap between conformational form and biological significance.

## Discovery of Zα domain recognizing Z-NAs

The functional significance of Z-NAs was first highlighted by the discovery of the Zα domain within adenosine deaminase acting on RNA 1 (ADAR1) [26]. Unlike traditional DNA-binding modules, the Zα domain employs a conformation-dependent but sequence-independent recognition strategy via its helix-turn-helix (HTH) motif [26]. Crystal structures show that two Zα domains bind to one Z-DNA duplex without any contact between the two Zα monomers [26].

In mammals, Z-DNA binding protein 1 (ZBP1/DAI/DLM-1) is the only other known protein possessing functional Zα domains besides ADAR1 [27]. Beyond mammals, Zα domains are found in the fish-specific protein kinase containing Zα domains (PKZ) [28] and the E3L protein of the vaccinia virus (VACV) [29]. While host-derived Z-NA binding proteins (ADAR1, ZBP1, and PKZ) converge on the regulation of immune responses, viral counterparts like E3L have evolved to subvert host defences [27–30]. Recent structure-based similarity searches and AlphaFold2 modelling have identified numerous candidates with high structural homology to the Zα domain [31]; however, their Z-NA binding capabilities and physiological relevance await experimental validation. Notably, a putative structural homolog of Zα—the Zτ domain, integrated into the catalytic core of DNA topoisomerase II – has been identified as a Z-NA binding module [32,33]. Although computational evidence suggests that Zτ enables the enzyme to function as a ‘conformase’, inducing B-to-Z transitions to stabilize topological subdomains during mitosis via an isomerase-inactive ZBP state [33], further *in vivo* validation is required to confirm the spatiotemporal dynamics and functional necessity of this interaction.

The identification of Zα domains as specific recognition modules has unlocked the structural mysteries underlying the biological activity of Z-NA ligands. However, Z-NA binding is merely the initiating event; its functional consequences are ultimately dictated by the conformational or allosteric changes induced in Zα-containing proteins upon ligation. Accumulating evidence demonstrates that this structural recognition event cascades into diversified intracellular signalling pathways. In ZBP1, Zα-mediated ligand engagement is coupled to RHIM-dependent signalling, driving the activation of inflammatory and programmed cell death pathways. Conversely, the Zα-containing p150 isoform of ADAR1 restrains the immunogenicity of endogenous dsRNA through RNA editing and Z-RNA regulation. These contrasting mechanisms provide a comprehensive framework for understanding how Z-NAs balance host defence and inflammation against nucleic acid immune tolerance. Consequently, the following sections will delineate the biological relevance of Z-NAs by focusing on their specific interactions with the Zα domains of ZBP1 and ADAR1.

## Molecular mechanisms and biological significance of Z-NA recognition by ZBP1

While the recognition of Z-NAs by Zα domains is well established, the mechanisms of immune modulation have only recently emerged through studies on the sensor protein ZBP1. ZBP1 is an interferon (IFN)-inducible protein whose N-terminus contains two Zα domains (Zα1 and Zα2) that confer specificity for Z-NAs [27]. ZBP1 primarily localizes in the cytoplasm but can shuttle between the cytoplasm and nucleus [34,35]. Initially, ZBP1 was identified as a B-DNA sensor that activates the TANK-binding kinase 1 (TBK1)/IFN regulatory factor 3 (IRF3) pathway via its C-terminus to induce type I IFNs [36]. However, primary cells obtained from ZBP1-deficient mice still produce type I IFNs in response to B-DNA or DNA virus infection [37], indicating that ZBP1 may be dispensable or functionally redundant in B-DNA-mediated responses. In addition, ZBP1 contains two canonical and one putative RIP homotypic interaction motifs (RHIMs), mediating interactions with RHIM-containing proteins such as receptor-interacting serine/threonine kinase 1 (RIPK1), RIPK3, and the TIR-domain-containing adaptor TRIF [38]. Through these interactions, ZBP1 can activate nuclear factor-κB (NF-κB) signalling and trigger apoptosis, necroptosis, and pyroptosis [39–41]. Below, we outline how Z-NAs activate ZBP1 and its downstream pathways under various physiological and pathological conditions.

### Viral infection

#### Cell death-dependent defence against RNA viruses

**Figure 1.**
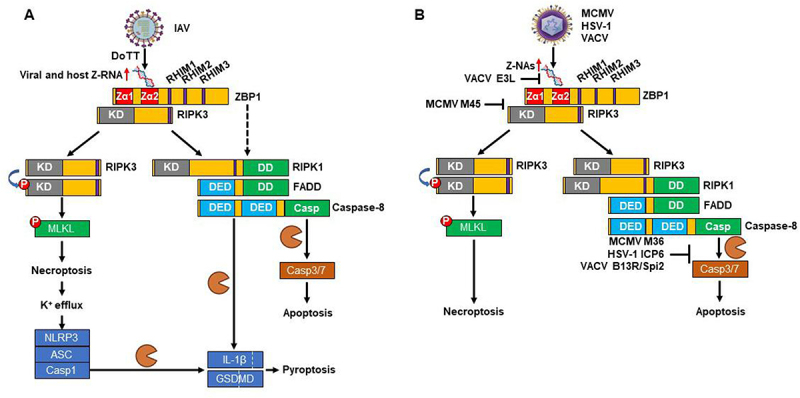
Viral Z-NA-mediated programmed cell death. (A) IAV infection triggers the accumulation of Z-RNA, derived from both viral genomes and host EREs via DoTT. These Z-RNAs activate ZBP1, which recruits RIPK3, thereby initiating divergent cell death pathways: MLKL-dependent necroptosis and FADD/caspase-8-mediated apoptosis. MLKL-induced membrane rupture facilitates K^+^ efflux, acting as a secondary signal for NLRP3-ASC-caspase-1 inflammasome assembly. This cascade leads to the proteolytic maturation of IL-1β and the cleavage of GSDMD, culminating in pyroptosis. Concurrently, caspase-8 can directly process GSDMD and pro-IL-1β to orchestrate pyroptotic cell death and cytokine release. (B) MCMV, HSV-1, and VACV infections trigger the accumulation of Z‑NAs – predominantly Z‑RNA. To counteract host defences, these viruses encode vICAs – such as MCMV M36, HSV-1 ICP6, and VACV B13R/Spi2—to block caspase-8-mediated apoptosis. Consequently, the accumulated Z-RNAs activate ZBP1, which recruits RIPK3 to orchestrate MLKL-dependent necroptosis. To evade necroptosis, MCMV encodes M45, which disrupts RHIM‑mediated ZBP1–RIPK3 interaction to block necroptosis. VACV encodes E3L, which competitively binds Z‑RNA, preventing its recognition by ZBP1.

ZBP1 plays a crucial role in antiviral defence by inducing programmed cell death, most notably during influenza A virus (IAV) infection. Upon IAV infection, Z-RNAs accumulate in the nucleus and become detectable by the Z22 antibody [25]. These Z-RNAs are primarily derived from defective viral genomes (DVGs) and host intergenic endogenous retroelements (EREs) [25,42]. The latter are incorporated into aberrant, elongated 3’ extensions triggered by the non-structural protein 1 (NS1)-mediated disruption of transcription termination (DoTT) [42]. Specifically, IAV NS1 sequesters the cleavage and polyadenylation specificity factor (CPSF), thereby impeding the canonical 3’ processing of nascent pre-mRNAs and forcing read-through transcription into normally silent genomic regions [42]. These Z-RNAs activate ZBP1 primarily via its Zα2 domain [40,43]. Once activated, ZBP1 engages RIPK3 through its RHIM1 motif to form the necrosome, leading to mixed lineage kinase domain-like protein (MLKL) phosphorylation, plasma membrane rupture, and necroptosis (Figure 1(A)) [39,40,43]. In parallel, RIPK3 can recruit RIPK1, which initiates Fas-associated protein with death domain (FADD) and caspase-8-dependentapoptosis through caspase-3/7 cleavage (Figure 1(A)) [39,40]. Although ZBP1 deficiency generally suppresses IAV-induced cell death and impairs viral control, *in vivo* studies report conflicting outcomes: one found decreased survival due to uncontrolled viral replication [40], while the other observed reduced lung damage and improved survival [39]. The basis for this discrepancy remains unclear. Although both apoptosis and necroptosis are detected in infected cell populations, they appear mutually exclusive at the single-cell level [44], likely due to heterogeneity in Z-RNA accumulation and protein expression. Additionally, IAV triggers NOD-like receptor family pyrin domain-containing protein 3 (NLRP3) inflammasome-mediated pyroptosis, which depends on prior apoptosis or necroptosis – either via MLKL-driven K^+^ efflux or via caspase-8-mediated cleavage of interleukin-1β (IL-1β) and gasdermin D (GSDMD) (Figure 1A) [45].

Solid arrows (→) denote activation, cleavage or induction; dashed arrow (⇢) denotes potential activation; T-shaped arrows (┤) denote inhibition; Symbols () denote proteolytic cleavage.

#### Cell death-dependent defence against DNA viruses

DNA viruses have evolved diverse strategies to evade infection-induced cell death. The DNA virus murine cytomegalovirus (MCMV) encodes protein M45, which contains an RHIM domain and acts as a viral inhibitor of RIP activation (vIRA) to suppress RIPK3-mediated necroptosis (Figure 1(B)) [46,47]. However, the M45 RHIM-mutant virus (MCMV-M45mutRHIM) triggers ZBP1-RIPK3-dependent cell death manifesting as MLKL-mediated necroptosis (Figure 1(B)), given that MCMV also encodes M36, a viral inhibitor of caspase-8 activation (vICA) that effectively suppresses extrinsic apoptosis (Figure 1(B)) [48]. *In vivo*, while wild-type (WT) mice are protected from MCMV-M45mutRHIM, mice lacking ZBP1, RIPK3, or MLKL suffer from uncontrolled viral replication and footpad swelling [43,47]. ZBP1 facilitates early viral clearance through its Zα domains, indicating that necroptosis triggered by infection-induced Z-NAs serves as a potent antiviral mechanism [43,49]. Notably, ZBP1 can mediate viral clearance in a Zα-independent manner during the late stage of infection [43], implying the involvement of additional mechanisms. Since newly transcribed viral RNA is required for ZBP1-mediated necroptosis during MCMV-M45mutRHIM infection [50], Z-RNA likely serves as the primary ligand for ZBP1 activation during MCMV-M45mutRHIM infection.

Herpes simplex virus-1 (HSV-1), another DNA virus belonging to the herpesviridae family, encodes the RHIM-containing protein infected cell protein 6 (ICP6), which displays a distinct species-specific bimodal effect: it directly activates RIPK3 to induce cell death in murine cells but antagonizes RIPK3 activation in human cells [51–53]. Beyond its RHIM-mediated functions, ICP6 also serves as a vICA to suppress apoptosis (Figure 1(B)) [54]. Interestingly, infection with ICP6-deficient or ICP6 RHIM-mutant HSV1 (HSV1ΔICP6 or HSV1(FmutRHIM)) triggers ZBP1-mediated necroptosis *in vitro* (Figure 1B) [55]. Consequently, the viral clearance mechanism is compromised in *Zbp1*^*-/-*^, *Ripk3*^*-/-*^, and *Mlkl*^*-/-*^ mice, allowing HSV1(FmutRHIM) to establish a productive infection [55]. The cell death induced by HSV-1(FmutRHIM) depends on the Zα domains of ZBP1, indicating that Z-NAs generated during infection activate ZBP1-dependent death [55]. Mechanistically, HSV-1 encodes ICP27, which triggers DoTT and the subsequent accumulation of host Z-RNA [42]. This process recapitulates the mechanism observed in IAV infection, wherein aberrant host transcripts serve as Z-RNA ligands to activate ZBP1 [42]. Notably, while HSV1(FmutRHIM)-induced necroptosis is primarily driven by RIPK3 in murine cells, the signalling pathway diverges in human cells, where the response predominantly relies on RIPK1-mediated recruitment of RIPK3 [56]. This variation likely stems from evolutionary structural divergence between human and murine RIPK3 RHIM domains [56].

Varicella zoster virus (VZV) also employs an RHIM-containing viral protein, open reading frame 20 (ORF20), to evade host defences [57]. ORF20 sequesters ZBP1 into amyloid-like aggregates, thereby antagonizing downstream signalling during infection [57]. In the absence of this inhibition – as seen with RHIM-mutant or deleted viruses (VZV-ORF20mutRHIM or VZV-ORF20ΔRHIM) – VZV specifically triggers ZBP1-mediated apoptosis rather than necroptosis in human cells to restrict viral replication [57].

DNA virus VACV, however, employs a distinct strategy to evade ZBP1-mediated cell death. It encodes E3L protein that contains both a Zα domain and a dsRNA-binding domain (dsRBD) [29]. During infection, the Zα domain competitively binds Z-RNA, thereby preventing ZBP1 activation (Figure 1(B)) [58]. VACV further inhibits cell death through the vICA B13R/Spi2 (Figure 1(B)) [59]. Therefore, infection with a mutant virus lacking the E3L Zα domain (VACV-E3LΔ83N) induces ZBP1-dependent necroptosis (Figure 1(B)) [58]. Consequently, VACV-E3LΔ83N establishes productive infection in *Zbp1*^*-/-*^ and *Ripk3*^*-/-*^ mice [58]. Unlike the nuclear Z-RNA induced by IAV, VACV-E3LΔ83N triggers cytoplasmic Z-RNA accumulation, demonstrating that Z-RNA from either cellular compartment can activate ZBP1 [60]. Intriguingly, during VACV infection, the dsRBD of E3L promotes the formation of Z-RNA, which contributes to ZBP1-mediated death signalling [60].

#### Cell death-independent defence against viruses

Conversely, during Zika virus (ZIKV) infection, compared with WT mice, mice lacking ZBP1 or RIPK3, as well as those expressing kinase-dead RIPK1 (*Ripk1*^K45A/K45A^), exhibit significantly higher viral titres in the central nervous system (CNS) after intracranial inoculation and increased mortality, whereas *Mlkl*^*-/-*^ mice do not – indicating that ZBP1 and RIPK3 function independently of the canonical necroptosis pathway [61]. Mechanistically, the ZBP1-RIPK1-RIPK3 axis induces immune-responsive gene 1 (IRG1) expression, whose metabolite itaconate promotes an antiviral metabolic state that suppresses ZIKV infection [61]. A similar protective role for ZBP1 is observed during West Nile virus (WNV) infection, with ZBP1-deficient mice also showing higher mortality [62]. Importantly, although both ZIKV and WNV replicate more efficiently in ZBP1-deficient neurons, infection in WT neurons does not induce overt cell death [61–63]. Together, these findings establish that ZBP1 can mediate a potent, cell death-independent antiviral defence within the CNS.

#### Cell death-mediated tissue damage

While the precise nature and biogenesis of Z-NAs elicited by VZV-ORF20 mutants, WNV, and ZIKV remain to be elucidated, SARS-CoV-2 infection has also been reported to induce robust Z-RNA accumulation, triggering ZBP1–RIPK3-mediated cell death [64]. However, this pathway fails to confer an antiviral advantage; instead, it exacerbates lung pathology and tissue damage – a deleterious phenomenon also observed during murine hepatitis virus (MHV) infection in mice [64,65].

In conclusion, Z-NAs – specifically Z-RNA – represent a conserved molecular trigger that activates ZBP1-mediated cell death, thereby orchestrating robust antiviral host defences. Emerging evidence further highlights the versatility of ZBP1, which can also initiate potent non-cytotoxic antiviral responses independent of programmed cell death. To counteract this, diverse viruses have evolved a sophisticated repertoire of evasion strategies to subvert or antagonize the ZBP1 signalling axis. Intriguingly, a recent study demonstrates that Z-DNA exhibits a markedly lower affinity for cGAS than B-DNA, suggesting that the B-to-Z transition may function as a molecular buffer to prevent excessive cGAS signalling [66]. This conformational shift is physiologically promoted by polyamines like spermine and spermidine [13]. Consequently, a deficiency in spermidine/spermine N1-acetyltransferase 1 (SAT1) – the rate-limiting enzyme in polyamine catabolism – alters polyamine flux and increases Z-DNA levels, thereby suppressing the cGAS pathway and modulating HSV-1 replication *in vivo* [66]. These findings highlight Z-DNA’s dual role as both a pro-inflammatory ZBP1 ligand and a cGAS-dampening decoy, a metabolic-epigenetic crosstalk that warrants further exploration.

### Ontogeny

**Figure 2.**
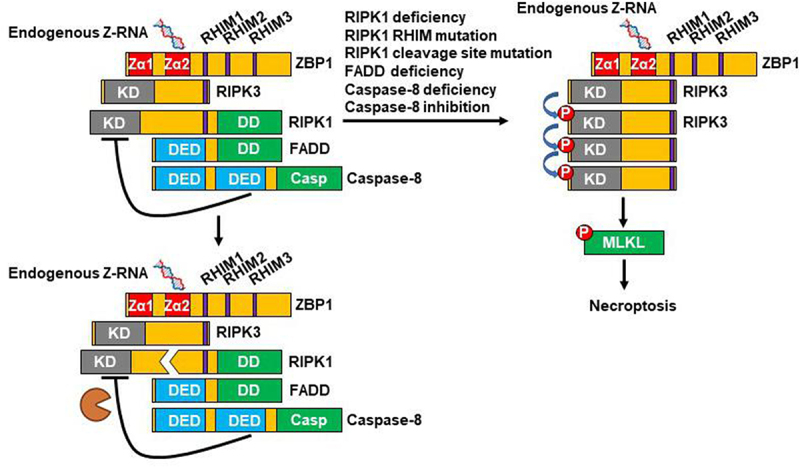
Suppression of basal Z-NA-driven necroptosis by the RIPK1-FADD-caspase-8 axis.

While the role of Z-NAs in viral host defence is well established, recent studies have uncovered significant infection-independent functions of endogenous Z-NAs. In mice, the ablation of RIPK1 leads to postnatal lethality driven by both FADD/caspase-8-mediated apoptosis and RIPK3/MLKL-mediated necroptosis [67,68]. ZBP1 serves as the critical trigger for this RIPK3 activation, as evidenced by the prolonged survival of *Ripk1*^*-/-*^*Caspase-8*^*-/-*^ mice upon ablation of ZBP1 [69,70]. To further elucidate how RIPK1 prevents aberrant ZBP1 activation, two independent RIPK1 RHIM-mutant (*Ripk1*^*mRHIM/mRHIM*^) mouse lines were generated to disrupt its interaction with other RHIM-containing proteins [69,71]. These mice displayed postnatal lethality that was completely rescued by ZBP1 ablation, confirming that RIPK1 utilizes its RHIM domain to constitutively inhibit ZBP1 [69,71]. Mechanistically, both the Zα domains (particularly Zα2) and the RHIM1 of ZBP1 are indispensable for driving this cell death [71]. Similarly, mutations in the RIPK1 death domain (DD, R588E) that abrogate DD-dependent interactions result in ZBP1-mediated perinatal lethality [72,73]. Consistent with these *in vivo* findings, cells lacking RIPK1 or those harbouring RHIM/DD mutations or RIPK1 cleavage-resistant variants exhibit heightened sensitivity to IFN-induced, ZBP1-dependent death (Figure 2) [43,70,72–75]. Collectively, these data provide robust evidence that endogenous Z-NAs activate ZBP1 to trigger lethal cell death when the inhibitory checkpoints of RIPK1 are compromised.

The recognition of endogenous Z-NAs via its Zα domains activates ZBP1, prompting its interaction with RIPK3. Under homoeostatic conditions, RIPK3 recruits RIPK1 and the FADD-caspase-8 complex; however, ZBP1-driven cell death is tonically inhibited by a negative regulatory loop. Within this complex, FADD-mediated recruitment of caspase-8 leads to the proteolytic cleavage of RIPK1, thereby preventing the aberrant activation of RIPK3. Consequently, in genetic or pharmacological contexts where this ‘brake’ is compromised – such as cells lacking RIPK1 or FADD, cells expressing cleavage-resistant or RHIM-mutant RIPK1, or those deficient in caspase-8 activity – the Zα-dependent sensing of endogenous Z-RNA triggers an unrestrained ZBP1-RIPK3 engagement, culminating in MLKL-dependent necroptosis.Figure adapted with permission from Ref [43].

Solid arrows (→) denote activation, cleavage or induction; T-shaped arrow (┤) denotes inhibition; Symbol () denotes proteolytic cleavage.

### Inflammation

#### Skin inflammation

The involvement of the Z-NA – ZBP1 axis in skin inflammation has been extensively characterized through conditional knockout models. Mice with an epidermis-specific RIPK1 deficiency (RIPK1^E-KO^) develop progressive inflammatory lesions defined by epidermal hyperplasia, aberrant differentiation marker expression, myeloid cell infiltration, and a robust surge in pro-inflammatory cytokines [76]. This pathology is strictly contingent upon ZBP1-mediated necroptosis, as the ablation of ZBP1, RIPK3, or MLKL confers complete protection [71]. Similarly, mice expressing RHIM-mutated RIPK1 (RIPK1^mRHIM/E-KO^) in the epidermis manifest a similar ZBP1-dependent necroptotic phenotype [71]. Notably, ZBP1 Zα mutations can rescue the skin inflammation phenotype in RIPK1^E-KO^ mice from 7 weeks to at least 18 weeks of age [43,77]. This rescue indicates that endogenous Z-NA sensing by ZBP1 plays a crucial role in driving skin inflammation in the absence of RIPK1. While functional Zα domains are crucial for initiating early-stage inflammation, their inactivation appears insufficient to maintain long-term protection in aged mice [43,77]. This suggests that ZBP1 also exerts Zα-independent functions in driving chronic skin inflammation, likely through its RHIM1 domain. This is supported by the finding that the ZBP1 RHIM1 mutation, much like total ZBP1 deficiency, effectively sustained protection against skin inflammation in aged RIPK1^E-KO^ mice, whereas Zα mutations did not [43]. Interestingly, mice express a truncated, alternatively spliced isoform, ZBP1-S, which retains the Zα domains but lacks the RHIMs. Functioning as an endogenous dominant-negative inhibitor, ZBP1-S competes for Z-NA ligands to prevent RIPK3 recruitment by the full-length protein [75,78]. Consequently, the loss of ZBP1-S exacerbates necroptosis-driven skin inflammation in RIPK1^E-KO^ mice [78], highlighting its essential role as a physiological brake that maintains epithelial homoeostasis. Similarly, the ZBP1-mediated necroptotic pathway has been implicated in the pathological progression of spontaneous skin inflammation in mice with epidermis-specific FADD deficiency (FADD^E-KO^) [79]. Emerging evidence identifies EREs as the source of putative ligands, with RNA-Seq confirming abundant ERE-derived dsRNA in the murine skin [43,80]. Collectively, these findings establish RIPK1 as an intrinsic homoeostatic checkpoint that prevents ZBP1-mediated skin inflammation from sensing endogenous Z-form dsRNA. Disruption of this regulatory mechanism may underlie the pathogenesis of chronic, early-onset inflammatory disorders in individuals harbouring *RIPK1* mutations [81–83]. In the presence of the intact RIPK1-FADD axis, the transgenic expression of constitutively active, C-terminally truncated ZBP1 (ZBP1ca) in the murine epidermis also triggers robust skin inflammation [84]. This pathology is only partially attenuated by the abrogation of RIPK3-MLKL-mediated necroptosis but is fully abolished by the combined deficiency of MLKL and caspase-8 [84]. Mechanistically, ZBP1ca orchestrates caspase-8-mediated apoptosis through a signalling axis that requires the RIPK1 RHIM domain but is independent of its kinase activity [84]. These findings demonstrate that, when RIPK1 is available, ZBP1 acts as a versatile scaffold to drive both RIPK3-dependent necroptosis and RIPK1-scaffold-dependent apoptosis. Beyond its regulation by RIPK1, the homoeostasis of mouse keratinocytes also requires the autophagic lipid scramblase autophagy-related protein 9A (ATG9A) [85]. Loss of ATG9A triggers severe dermatitis and systemic inflammation, a pathology orchestrated by cGAS/stimulator of IFN genes (STING)-dependent Type I IFN production [85]. This IFN signalling primes subsequent ZBP1-dependent apoptosis and necroptosis, presumably by facilitating the sensing of epidermal Z-DNA [85]. These findings establish a mechanistic link between autophagic lipid scrambling and the ZBP1-mediated inflammatory response.

#### Intestinal inflammation

Beyond skin pathology, Z-NAs also serve as potent drivers of intestinal inflammation. Mice with an intestinal epithelial cell-specific FADD deficiency (FADD^IEC-KO^) develop spontaneous colitis and ileitis, characterized by epithelial hyperplasia, ulcerating lesions, Paneth cell loss, and robust immune cell infiltration – all of which stem from aberrant RIPK3 activation [86]. In this model, the progression of ileitis is significantly abrogated only by the concurrent ablation of tumour necrosis factor receptor 1 (TNFR1) and ZBP1 [87]. In contrast, the loss of either ZBP1 or TNFR1 alone is sufficient to prevent colitis [87], suggesting that these two pathways synergistically drive colonic pathology but function redundantly in the small intestine. Crucially, FADD^IEC-KO^ mice harbouring ZBP1 Zα domain mutations are strongly protected from colonic inflammation, establishing that endogenous Z-NAs are the requisite ligands for aberrant ZBP1 activation in the gut [43]. Furthermore, the observation that FADD/caspase-8 deficiency or inhibition sensitizes cells to ZBP1-mediated necroptosis identifies the FADD – caspase-8 axis as a checkpoint restraining this pathway (Figure 2) [43,49,74,87]. Interestingly, this ZBP1-dependent cell death is also contingent upon RIPK1 kinase activity [43,49,87], although the precise underlying mechanisms remain to be elucidated. This mechanism likely underlies the inflammatory phenotypes observed in patients with *CASP8* mutations [88]. Integrated with developmental and skin data from RIPK1-mutant mice, these studies define a critical RIPK1–FADD – Caspase-8 regulatory hub that negatively modulates ZBP1-mediated necroptosis and inflammation triggered by endogenous Z-NAs (Figure 2). Another study also identified aberrant ZBP1 activation in the gut following tamoxifen-induced deletion of SET domain bifurcated histone lysine methyltransferase 1 (SETDB1) gene in intestinal epithelial cells (SETDB1^iIEC-KO^) [80]. As a histone methyltransferase, SETDB1 maintains genomic integrity by mediating histone H3 lysine 9 trimethylation (H3K9me3), a modification essential for the transcriptional silencing of EREs. Upon tamoxifen induction, SETDB1^iIEC-KO^ mice manifest fulminant ileitis and colitis, characterized by severe villous atrophy, crypt and colonic deformation, and massive immune cell infiltration, ultimately resulting in mortality [80]. Notably, the ablation of ZBP1, RIPK3, or MLKL significantly delays disease progression and reduces mortality, establishing ZBP1-mediated necroptosis as a primary driver of the intestinal pathology [80]. *In vitro* assays further demonstrate that ZBP1 directly binds ERE-derived dsRNA in *Setdb1*^−/−^ intestinal stem cell (ISC)-derived spheroids [80]. Crucially, the clinical relevance of this mechanism is underscored by findings in inflammatory bowel disease (IBD) patients, who exhibit decreased SETDB1 expression alongside elevated ZBP1 expression and MLKL phosphorylation levels [80]. Collectively, these data uncover a novel pathological role for ZBP1 in IBD, where it functions as a sensor for de-repressed EREs. Moreover, recent research has identified a specialized IFN-λ/ZBP1/GSDMC axis that restricts intestinal repair [89]. Upon sensing injury-induced Z-NAs, ZBP1 triggers a caspase-8–GSDMC pyroptotic pathway that halts epithelial regeneration [89].

#### Other inflammatory disorders

**Figure 3.**
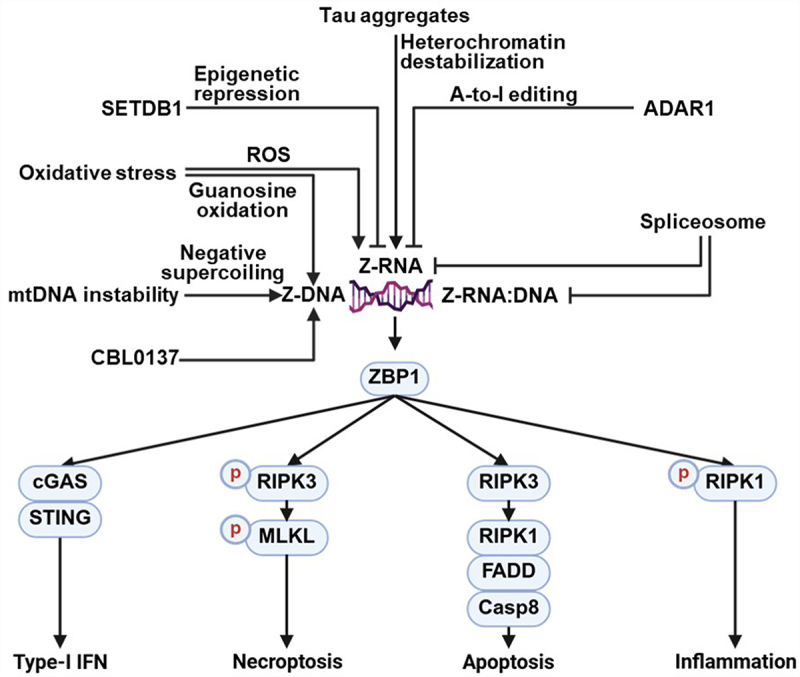
Mechanisms regulating Z-NAs abundance and downstream signalling.

Mitochondrial DNA (mtDNA) has emerged as a pivotal source of Z-DNA, which acts as an endogenous ligand that primes ZBP1-mediated inflammatory cascades across various tissues. Recent findings elucidate that amyloid-β (Aβ)-induced oxidative stress promotes the fragmentation and cytoplasmic release of mtDNA, which adopts a Z-conformation upon oxidation (Figure 3) [90]. In Alzheimer’s disease (AD)-associated microglia, ZBP1 acts as a sentinel for this oxidized Z-DNA via its Zα domains, subsequently recruiting RIPK1 to activate its kinase-dependent pro-inflammatory signalling [90]. This ZBP1–RIPK1 neuroinflammatory cascade exacerbates Aβ deposition and cognitive decline, while the genetic ablation of ZBP1 or pharmacological inhibition of RIPK1 kinase activity effectively mitigates these pathologies [90]. Beyond microglial inflammation, tau aggregates drive neurodegeneration by sequestering H3K9me3 to destabilize heterochromatin, thereby triggering the transcriptional reactivation of ERE-derived Z-RNAs that elicit ZBP1-mediated neuronal death [91,92]. Together, these insights identify the sensing of Z-NAs as a key innate immune trigger in neurodegeneration, positioning ZBP1 as a promising therapeutic target for neurodegenerative diseases.

The compound CBL0137 stimulates the formation of Z-DNA in the nucleus. In mitochondria, oxidative stress causes oxidation of mtDNA, driving its transition into Z‑DNA – a process also facilitated by mitochondrial genomic instability, which induces negative supercoiling of mtDNA. Oxidative stress promotes Z‑RNA formation via reactive oxygen species (ROS). ADAR1 in the cytoplasm or SETDB1 in the nucleus prevents Z‑RNA accumulation through RNA editing or epigenetic repression, respectively. Tau aggregates cause destabilization of heterochromatin, leading to EREs reactivation and Z-RNA accumulation. Splicing inhibition or spliceosome mutation contributes to the formation of Z‑RNA:DNA hybrids and Z‑RNA. Accumulated Z‑NAs activate ZBP1, which recruits RIPK3 to initiate distinct cell death pathways: MLKL-dependent necroptosis and FADD/caspase‑8-mediated apoptosis. ZBP1 activation can also trigger RIPK1 kinase-dependent inflammatory responses. Furthermore, ZBP1 synergizes with cGAS to amplify cGAS – STING-driven type I IFN responses.

Solid arrows (→) denote activation or induction; T-shaped arrow (┤) denotes inhibition.

The pathological significance of the mitochondrial Z-DNA – ZBP1 axis extends beyond the central nervous system. For instance, acetaminophen overdose induces the accumulation of 8-oxoG-modified mtDNA, which undergoes an oxidative stress-driven B-to-Z conformational transition [17]. These Z-form mtDNA fragments subsequently leak into the cytosol, where they serve as endogenous ligands to trigger ZBP1-dependent hepatocyte death and acute liver failure [17]. Similarly, ultraviolet (UV)-induced damage precipitates mitochondrial stress, triggering the cytosolic extrusion and oxidation of mtDNA [93]. This oxidized mtDNA adopts the Z-conformation that is stabilized by ZBP1, which potentiates the cGAS – STING-mediated Type I IFN response [93]. This ZBP1-driven feed-forward loop exacerbates cutaneous autoinflammation and underlies the pathogenesis of photosensitivity in skin cells [93]. Anthracycline-induced cardiotoxicity, as exemplified by doxorubicin (Doxo), stems from mitochondrial genome instability. A recent study demonstrated that Doxo promotes mtDNA supercoiling and generates torsional strain – a pathological state exacerbated by DNA topoisomerase I mitochondrial (TOP1MT) deficiency – which subsequently triggers the cytosolic release of mtDNA [94]. Once in the cytosol, the displaced mtDNA is stabilized in a Z-conformation by ZBP1, leading to the assembly of a cGAS – RIPK1–RIPK3 signalling hub that perpetuates a chronic type I IFN response [94]. This persistent immune activation, driven by ZBP1-mediated co-sensing of mtDNA, ultimately underlies the progression of cardiomyocyte dysfunction and heart failure [94].

The functional role of ZBP1 in lipopolysaccharide (LPS)-induced sepsis – a life-threatening systemic inflammatory response – remains a subject of debate due to conflicting experimental outcomes in mouse models. One study demonstrated that ZBP1 potentiates TRIF-dependent inflammatory signalling through a mechanism independent of its Z-NA sensing capacity, thereby exacerbating septic shock [95]. In contrast, other research indicates that ZBP1 serves a protective role by dampening TRIF-mediated necroptosis, specifically within autophagy-deficient backgrounds [96]. These disparate findings not only suggest that the influence of ZBP1 on septic progression is highly context-dependent – potentially modulated by the cell’s underlying proteostatic or autophagic status – but also imply ZBP1 may function as a broader pathological mediator through ligand-independent mechanisms, extending beyond its canonical role as a nucleic acid sensor. Further investigation is warranted to reconcile these divergent observations.

### Stress-driven pathology

In the context of heatstroke, a systemic crisis defined by rapid hyperthermia and multi-organ failure, ZBP1 has been identified as a requisite driver of RIPK3-dependent necroptosis [97]. Notably, heat stress-induced ZBP1 activation does not require Z-NAs but is instead initiated by ZBP1 aggregation via its RHIM1 domain *in vitro* [97]. By contrast, several other physiological or environmental stressors that promote stress-granule formation have been proposed to trigger the accumulation of ERE-derived Z-RNA to activate ZBP1-dependent necroptosis via its Zα domains (Figure 3) [98–100]. Therefore, the role of Z‑NA sensing in the pathogenesis of heatstroke *in vivo* remains to be validated. This paradigm of ZBP1-driven, stress-responsive necroptosis extends to male infertility, particularly non-obstructive azoospermia (NOA) [101]. Within the testis, the assembly of the ZBP1–RIPK3 axis triggers MLKL phosphorylation, leading to the necroptotic demise of spermatogonia and Sertoli cells [101]. The physiological significance of this axis is underscored by the finding that genetic ablation of ZBP1 or RIPK3 confers protection against heat-induced testicular degeneration [101]. Crucially, the activation of this necroptotic pathway is also observed in aged human testes [101], suggesting that this stress-coupled death mechanism contributes to both pathological infertility and the broader process of reproductive ageing. However, the requirement for Z-NA sensing in this context remains to be experimentally validated.

### Tumorigenesis and intervention

ZBP1 has emerged as a central integrator of endogenous stress signals that suppresses oncogenesis and tumour progression. For example, in response to telomere dysfunction, the cGAS – STING pathway upregulates a crisis-specific human ZBP1 isoform that lacks Zα1 [102]. This isoform senses telomeric repeat-containing RNA (TERRA) via its Zα2 domain and assembles into filaments on the outer mitochondrial membrane, where it activates the adaptor mitochondrial antiviral signalling protein (MAVS) to drive a potent IFN response that eliminates premalignant cells before they achieve immortality [102]. Beyond telomere surveillance, ZBP1 also helps maintain genomic integrity, presumably by detecting accumulated Z-RNA and Z-DNA during neoplastic transformation, thereby linking meiotic recombination 11 (MRE11) – cGAS – detected DNA double-strand breaks to the RIPK3–MLKL necroptotic axis to restrain oncogenic proliferation [103]. ADAR1 acts as a molecular checkpoint that sequesters ZBP1 to promote immune evasion; genetic deletion of ADAR1 significantly impairs colorectal cancer and melanoma progression [99]. These findings highlight this pathway as a promising therapeutic target. Pharmacological agents such as curaxin CBL0137, nuclear export inhibitors, and spliceosome inhibitors can potently trigger ZBP1-dependent cell death via Zα domains sensing Z-DNA, Z-RNA, or Z-RNA:DNA (Figure 3) [43,104–108]. Whether administered as monotherapies or in synergy with anti – programmed cell death protein 1 (PD-1) blockade, these compounds orchestrate the conversion of immunologically ‘cold’ tumours into ‘hot’ ones by fostering an immunogenic microenvironment [104,105,108]. While tumour cells typically downregulate RIPK3 to escape necroptosis [109–111], ZBP1 can induce regulated death in cancer-associated fibroblasts (CAFs), leading to the release of pro‑inflammatory mediators that stimulate systemic anti‑tumour immunity [105]. Notably, an emerging paradigm proposes that Z-RNA:DNA hybrids represent a novel class of immunogenic ligands under conditions of spliceosome inhibition [106]. These hybrids are likely derived from R-loop structures, in which the nascent RNA transcript re-anneals with the DNA template, thereby displacing the non-template strand. Evidence from R-loop ChIP (R-ChIP) confirms the *in vivo* occurrence of these non-canonical structures [112]. While their exact architectural topology remains to be fully elucidated, these Z-RNA:DNA hybrids may function as potent substrates for ZBP1 recognition, triggering a cell death cascade that contributes to the pathogenesis of myelodysplastic syndromes (MDS) driven by mutations in splicing factors such as *SF3B1*, *U2AF1*, and *SRSF2* [113]. Collectively, these findings redefine ZBP1 as a versatile stress rheostat that coordinates multiple tumour‑suppressive barriers ranging from telomere maintenance and genomic fidelity to dynamic modulation of the tumour microenvironment. Together with mechanisms in SETDB1-mutant inflammatory disease, these findings reveal a common regulatory layer emerges: the pathological accumulation of endogenous Z-NA ligands (Figure 3). This ligand ‘overload’ hyperactivates the ZBP1 axis, driving aberrant cell death and systemic inflammation (Figure 3).

Collectively, Z-NAs appear to function by activating ZBP1 to trigger cell death, thereby mediating antiviral, anti-tumour, and pro-inflammatory effects. Importantly, this relationship is not exclusive: Z-NAs can also activate ZBP1 to elicit cell death-independent biological functions, whereas ZBP1 itself can induce cell death through Z-NA-independent mechanisms.

## Molecular mechanisms and biological significance of Z-NA recognition by ADAR1

The RNA-editing enzyme ADAR1 is the only other mammalian protein known to harbour a Zα domain, sharing this rare structural motif with ZBP1 [114]. ADAR1 is expressed as two distinct isoforms: the constitutively active p110, which is predominantly sequestered in the nucleus, and the interferon-inducible p150, which is primarily cytoplasmic [114]. While both isoforms share a catalytic deaminase domain – responsible for adenosine-to-inosine (A-to-I) editing – alongside three dsRBDs and a Zβ domain, only the p150 isoform possesses the N-terminal Zα domain [114]. Notably, although the Zβ domain adopts a structural fold nearly identical to Zα, it lacks the critical amino acid residues required for Z-NA coordination, rendering it incapable of recognizing Z-NAs [114].

**Figure 4.**
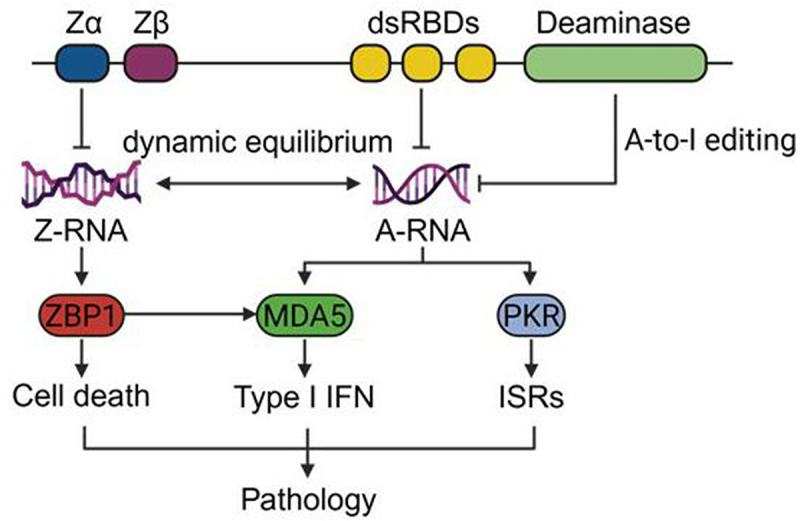
Mechanisms underlying ADAR1-mediated suppression of autoinflammation.

*ADAR1* mutations are a major genetic cause of Aicardi – Goutières syndrome (AGS), a severe hereditary encephalopathy characterized by early-onset neuroinflammation and systemic autoimmunity [115]. Initially recognized for its resemblance to congenital viral infections, AGS is now understood as a monogenic interferonopathy resulting from defects in cellular nucleic acid metabolism [115]. Mechanistically, ADAR1 preserves self-tolerance by catalysing adenosine-to-inosine (A-to-I) editing of endogenous dsRNAs, particularly those derived from EREs such as Alu elements and other short interspersed nuclear elements (SINEs) [114]. This editing prevents the aberrant activation of the MDA5–MAVS – IFN signalling axis and inhibits the protein kinase R (PKR)-driven integrated stress response (Figure 4) [116]. Genetically, the majority of reported ADAR1‑associated AGS cases harbour compound heterozygous mutations, most frequently combining the P193A missense mutation in the Zα domain with a loss‑of‑function or deaminase‑domain mutation [115,117]. To unravel the pathophysiology of these clinical variants, a range of ADAR1‑deficient and ADAR1‑mutant mouse models have been developed, yielding important insights into the roles of ADAR1 in maintaining RNA homoeostasis.

ADAR1 employs multiple functional domains – including its Zα domain, dsRBDs, and deaminase domain – to restrict the accumulation of both endogenous A‑RNA and Z‑RNA. Through this coordinated activity, ADAR1 acts as a critical gatekeeper against autoinflammatory pathology, suppressing the activation of distinct downstream immune pathways: it blocks MDA5‑mediated type I IFN responses and PKR‑driven ISRs triggered by A‑RNA while simultaneously inhibiting ZBP1‑dependent cell death and inflammation induced by Z‑RNA.Figure adapted with permission from Ref [105].

Solid arrows (→) denote activation or induction; T-shaped arrows (┤) denote inhibition.

### Adar1 loss-of-function mouse models

The analysis of genetically engineered mouse models reveals the essential and complex roles of ADAR1 in development and homoeostasis. Complete ADAR1 knockout (*Adar1*^−/−^) leads to embryonic lethality by embryonic day 12.5 (E12.5), characterized by type I interferon overproduction, haematopoietic failure, and widespread cell death, phenotypes that are rescued by co-deficiency of MDA5 or its adaptor MAVS, but not by loss of IFN alpha and beta receptor subunit 1 (IFNAR1), STING, or RIG-I [118–120]. This demonstrates that ADAR1 primarily prevents lethal MDA5-MAVS pathway activation by editing endogenous dsRNA. Interestingly, while MAVS deletion or MDA5 deletion rescues embryonic lethality, *Adar1*^−/−^*Mavs*^−/−^ mice and *Adar1*^−/−^*Mda5*^−/−^ mice succumb postnatally to severe intestinal and kidney pathology [120]. The lethality of *Adar1*^−/−^*Mavs*^−/−^ is driven by ZBP1, as ablation of ZBP1 or mutation of its Zα domains, or deletion of the necroptosis mediator RIPK3 gene, allows *Adar1*^−/−^*Mavs*^−/−^ mice to survive to adulthood [121,122]. On the other hand, the lethality of *Adar1*^−/−^*Mda5*^−/−^ is driven by PKR, as deletion of PKR gene allows *Adar1*^−/−^*Mda5*^−/−^ mice to survive to adulthood [116]. This indicates ADAR1 prevents MDA5-MAVS-mediated pathogenic IFN responses, ZBP1-mediated cell death, and PKR-mediated innate immune responses (Figure 4). Isoform-specific knockouts further delineate these functions. Mice lacking the IFN-inducible p150 isoform (*Adar1*^p150-/p150-^) phenocopy the embryonic lethality of full knockout, which is also rescued by MDA5/MAVS deficiency and PKR deficiency, confirming p150 as the key suppressor of the MDA5 and PKR pathway [116,120]. In contrast, mice lacking the constitutive p110 isoform (*Adar1*^p110-/p110-^) display partial postnatal lethality and growth retardation but no overt inflammation, suggesting p110 has distinct, developmentally important roles unrelated to innate immune suppression [123]. Notably, kidney defects in *Adar1*^−/−^*Mavs*^−/−^ mice are absent in *Adar1*^p150-/p150-^*Mavs*^−/−^ mice, implicating the role of p110 in protecting against developmental kidney pathology [120]. Similarly, mice harbouring a catalytically inactive ADAR1 deaminase domain (*Adar1*^E861A/E861A^) also die during embryogenesis in an MDA5-dependent manner, confirming that RNA-editing activity is essential for preventing aberrant MDA5 activation (Figure 4) [124]. Strikingly, these editing-deficient mice, like complete *Adar1* knockouts, are rescued to adulthood by MDA5 co-deficiency [124], resembling the rescue observed in *Adar1*^−/−^*Mavs*^−/−^*Zbp1*^−/−^ mice and *Adar1*^−/−^*Mda5*^−/−^*Pkr*^−/−^ mice, which suggests ADAR1’s Zα domain or dsRBDs may independently restrain ZBP1 and PKR (Figure 4). Collectively, these models establish ADAR1 as a pivotal guardian of cellular homoeostasis, functioning as a checkpoint that prevents unedited A-form dsRNA from MDA5 and PKR detection and Z-form RNA from ZBP1 recognition (Figure 4).

### Adar1 Zα domain-mutant mouse models

To specifically investigate the role of ADAR1’s Zα domain, researchers have studied mice carrying point mutations within this region. Proline 193, the most frequently mutated residue within the human ADAR1 Zα domain, directly contacts the characteristic left-handed helical structure of Z-NAs and is essential for the ADAR1p150-Z-NA interaction [26]. Consequently, the P193A (P195A in mouse) mutation diminishes this binding affinity, leading to significantly reduced dsRNA editing efficiency [119]. Mice homozygous for the P195A mutation (*Adar1*^P195A/P195A^) are phenotypically normal, explaining the lack of reported homozygous patients [125]. However, modelling AGS with compound genotypes (*Adar1*^P195A/-^ or *Adar1*^P195A/p150-^) recapitulates disease mortality and growth defects [125]. This pathology is driven by the MDA5 pathway, as MDA5 deficiency completely rescues viability [125]. Detailed analysis of *Adar1*^P195A/p150-^ mice revealed MDA5- and IFNAR1-dependent organ damage in the kidney, liver, and spleen, which is critically dependent on PKR-mediated integrated stress responses (ISRs) [125]. Because the P195A mutation resides in the Zα domain, it suggests a functional link to ZBP1. Consistent with this idea, ZBP1 deficiency largely rescued the phenotype of these mice. Notably, however, this rescue occurred through a cell death – independent mechanism, as deficiency of RIPK3 or MLKL, or inactivation of RIPK1 kinase activity, provided little benefit [126]. Complementary insights come from mice with complete loss of Z-NA binding (N175D/Y179A or N175A/Y179A), hereafter referred to as *Adar1*^mZα/mZα^ mice. These mice are viable but show mild, MAVS- and ZBP1-dependent IFN signatures, which confer a potent protective effect against viral infections [122,127,128]. The compound *Adar1*^mZα/-^ genotype, however, causes severe postnatal lethality with gut cell death and anaemia, primarily rescued by MAVS deficiency [121,122]. ZBP1 deficiency also significantly improves survival, independent of canonical necroptotic or apoptotic pathways mediated by RIPK3/MLKL and FADD/caspase-8, reinforcing a cell death-independent pro-inflammatory role for ZBP1 in this context [121,122]. In contrast, mice with the W197A mutation (*Adar1*^W197A/W197A^), affecting another critical Z-DNA interaction residue, exhibit severe multi-organ developmental defects and brain malformations resembling human AGS encephalopathy [129]. This severe phenotype is entirely MDA5-dependent and is not mitigated by the mutant protein’s residual, and even feedback-enhanced, RNA-editing activity, indicating that the Zα domain’s structural integrity is essential for preventing MDA5-mediated inflammation during development [129]. Collectively, these genetic models delineate a dual safeguarding mechanism: ADAR1’s Zα domain is structurally critical to prevent MDA5 activation (as seen with W197A), while its Z-RNA binding function, when compromised (as in P195A or mZα models), unmasks a parallel, cell death-independent pro-inflammatory pathway mediated by ZBP1. The PKR-ISR axis emerges as a key driver of the resulting tissue damage, highlighting a potential therapeutic target for ADAR1-linked disorders.

Although this interferonopathy in *Adar1*^*mZα/–*^ mice is independent of FADD-mediated apoptosis and MLKL-mediated necroptosis, in cell culture, ADAR1 deficiency leads to Z-RNA accumulation and ZBP1-dependent cell death (Figure 4) [105]; deeper analysis identified two sequence types capable of forming Z-RNA in ADAR1-deficient cells: SINEs and GU-type simple repeats [105]. Consistently, RNA-seq shows substantial upregulation of EREs and reduced RNA editing in *Adar1*^*mZα/–*^ tissues [122], leading to accumulation of dsRNA, which forms putative Z-RNA ligands that promote a ZBP1-mediated pathogenic IFN response. Moreover, transfection of *in vitro*-transcribed Alu elements induces ZBP1-mediated cell death [121], further supporting unedited ERE-derived Z-RNA as a natural ZBP1 ligand that can trigger cell death and pathology (Figure 4). The underlying cell death-independent role of ZBP1 in promoting pathogenic IFN responses remains to be further investigated.

## Concluding remarks

Since their discovery in the 1970s, Z‑NAs – including Z‑DNA, Z‑RNA, and Z‑RNA:DNA hybrids – have received far less attention than their canonical double‑stranded counterparts. In recent years, however, they have emerged not merely as structural curiosities but as pivotal regulators of innate immunity and inflammatory disease. Through its Zα domains, ZBP1 detects endogenous Z‑NAs and can trigger either RIPK3‑dependent cell death or RHIM‑mediated inflammatory responses, thereby shaping antiviral defence and inflammatory pathology. Yet how Z‑NA binding to Zα activates downstream RHIM signalling remains largely undefined. Recent work suggests that Z‑NA accumulation promotes liquid – liquid phase separation (LLPS) and oligomerization of Zα domains [130,131], pointing to biomolecular condensation as a potential mechanism for ZBP1 activation.

Notably, Z‑NA functions extend well beyond ZBP1 signalling. During bacterial infection, Z‑DNA within biofilms helps maintain structural integrity and confers resistance to DNase degradation [132,133]. In male germ cell development, the protein zinc finger and BTB domain-containing protein 43 (ZBTB43) remodels Z‑DNA to prevent DNA breaks and mutations, thereby safeguarding genetic transmission [134]. Genetic models and human disorders underscore the dual nature of Z‑NAs: protective in infection but pathogenic when dysregulated – a duality whose mechanisms demand deeper exploration. Given their involvement across diverse pathologies, Z‑NAs may serve not only as molecular markers of cellular stress but also as actionable therapeutic targets in virology, immunology, and oncology. Future studies should therefore aim to elucidate their context‑dependent roles and develop strategies to selectively modulate Z‑NA recognition or turnover, opening new avenues for clinical intervention.

## Data Availability

No data availability statement.
